# Tertiary Lymphoid Organs in Central Nervous System Autoimmunity

**DOI:** 10.3389/fimmu.2016.00451

**Published:** 2016-10-25

**Authors:** Meike Mitsdoerffer, Anneli Peters

**Affiliations:** ^1^Klinikum Rechts der Isar, Department of Neurology, Technical University Munich, Munich, Germany; ^2^Munich Cluster for Systems Neurology (SyNergy), Munich, Germany; ^3^Department of Neuroimmunology, Max Planck Institute of Neurobiology, Martinsried, Germany

**Keywords:** multiple sclerosis, EAE, TLO, ectopic lymphoid follicles, autoimmunity

## Abstract

Multiple sclerosis (MS) is an autoimmune disease characterized by chronic inflammation in the central nervous system (CNS), which results in permanent neuronal damage and substantial disability in patients. Autoreactive T cells are important drivers of the disease; however, the efficacy of B cell depleting therapies uncovered an essential role for B cells in disease pathogenesis. They can contribute to inflammatory processes *via* presentation of autoantigen, secretion of pro-inflammatory cytokines, and production of pathogenic antibodies. Recently, B cell aggregates reminiscent of tertiary lymphoid organs (TLOs) were discovered in the meninges of MS patients, leading to the hypothesis that differentiation and maturation of autopathogenic B and T cells may partly occur inside the CNS. Since these structures were associated with a more severe disease course, it is extremely important to gain insight into the mechanism of induction, their precise function, and clinical significance. Mechanistic studies in patients are limited. However, a few studies in the MS animal model experimental autoimmune encephalomyelitis (EAE) recapitulate TLO formation in the CNS and provide new insight into CNS TLO features, formation, and function. This review summarizes what we know so far about CNS TLOs in MS and what we have learned about them from EAE models. It also highlights the areas that are in need of further experimental work, as we are just beginning to understand and evaluate the phenomenon of CNS TLOs.

## Introduction

Multiple sclerosis (MS) is a chronic inflammatory disease of the central nervous system (CNS). Based on the disease course, three different subtypes of MS are distinguished. The vast majority of patients exhibit a relapsing-remitting disease course (RRMS) at first. However, after a variable period of time (usually 10–15 years), RRMS patients experience progressive disability independent of relapses. Hence, this subtype is called secondary progressive MS (SPMS). Few patients (about 10%) experience a progressive disease course from the beginning [primary progressive MS (PPMS)] (1).

Demyelination, axonal damage, and neuronal loss as a result of immune cell infiltration are hallmarks of the disease. A large body of evidence supports the concept of MS being a T cell-mediated autoimmune disease presumably directed against CNS antigens (2). However, it is not clear yet whether lesion formation requires additional immune mechanisms, such as antibody-mediated tissue destruction (3).

Intrathecal production of oligoclonal antibodies is a characteristic feature in MS patients (4). Moreover, plasma cells (PCs) accumulate in lesions and cerebrospinal fluid (CSF). Detection of immunoglobulin G (IgG) and activated complement fragments and complexes in close proximity to actively demyelinating lesions indicates the involvement of antibody-mediated effector mechanisms. Technical progress over the last decade has helped to further characterize the humoral immune response in MS patients. Detection of extensive somatic hypermutation and a high frequency of clonally expanded memory B cells in MS lesions and CSF suggest that the observed B cell response is antigen-specific and not a random bystander effect (5).

However, recent clinical trials with a B cell depleting agent (anti-CD20 antibody) indicate that B cells are significant in the pathogenesis of MS beyond the production of antibodies (6). Anti-CD20 therapy rapidly reduces recurrence of relapses in MS patients, while antibody levels in the CSF remain essentially unaffected (7). B cells may also act as efficient antigen-presenting cells (APCs), especially in the context of cognate B cell:T cell interactions (8). Furthermore, the release of pro- (IL-6, LTα, TNFα, GM-CSF) and anti-inflammatory (IL-10, IL-35) cytokines by B cells may affect the immune response. Interestingly, analysis of B cells in MS patients suggests a preponderance of pro-inflammatory cytokines (9).

In this context, it was a significant finding that a relevant fraction of SPMS patients show B cell-rich meningeal immune cell collections. These structures recapitulate lymphoid follicle-like features to some extent and, therefore, may provide an excellent microenvironment for the interaction of B and T cells (10, 11).

Initiation of adaptive immune responses as well as maintenance of immune homeostasis requires tightly regulated processes in our immune system. Secondary lymphoid organs (SLO) constitute an important platform of this sophisticated system (12). The architecture of SLOs allows intense interactions between the different cellular components and, therefore, facilitates antigen presentation to T and B cells. Encapsulated SLOs develop under the control of precise genetic programs at predefined key locations in the body during embryonic development and include the spleen and the lymph nodes. Those structures allow monitoring of self and foreign antigens displayed by APCs that survey tissues and traffic to SLOs. Lymphoid organs exhibit several typical features that allow the generation of a fast and efficacious anti-pathogen response. Germinal centers constitute one of them. These highly organized structures bring together antibody-secreting and proliferating B cells and follicular dendritic cells (FDCs) (13). Further characteristics are a T-cell zone populated by naïve T cells and central memory T cells recruited from the blood; high endothelial venules (HEV); and a network of stromal cells that provide chemokines and extracellular matrix (ECM) for cellular migration and structural integrity (14).

Inappropriate control of the immune system, for example, during autoimmunity, results in chronic inflammation. Under these circumstances, immune cells that infiltrate into peripheral tissues can shape highly organized follicle-like structures that share various features of SLOs. Accordingly, these ectopic lymphoid follicles are called tertiary lymphoid organs (TLOs) (12). Similarities include the presence of T and B compartmentalization, presence of APCs such as dendritic cells (DCs) and FDCs, stromal cells, HEV, and lymphatic vessels. However, in contrast to SLOs most TLOs lack a capsule. Moreover, TLOs are transient structures that often disintegrate upon clearance of the antigen.

Tertiary lymphoid organs have been observed in various forms of chronic inflammation, such as autoimmunity, chronic graft rejections, persistent infection, artherosclerosis, and cancer (12, 15–17).

Various factors of the local tissue microenvironment are involved in this process. Certain inflammatory mediators, such as members of the lymphotoxin (LT) family as well as different cytokines induce TLOs (18). Moreover, certain subsets of immune cells have been implicated in the development of TLOs (18). Even though their precise role is still elusive, it is assumed that they act as local sites of antigen presentation and lymphocyte activation, including somatic hypermutation and class switch recombination in B cells. Therefore, it stands to reason that they provide the ideal environment for antimicrobial responses, epitope spreading, as well as autoimmune exacerbation.

This review will discuss the occurrence and significance of TLOs in MS and its animal model experimental autoimmune encephalomyelitis (EAE).

## Occurrence and Significance in MS

### Features/Characteristics of TLOs in MS

A morphological study in the late 1970s described accumulations of immune cells in chronic MS lesions (19). The authors found lymphocytes, macrophages, and PCs interacting with reticular cells in the perivascular space of parenchymal blood vessels (19). In addition, recent studies discovered distinct inflammatory infiltrates around blood vessels of the leptomeninges of MS patients, in particular in SPMS patients at a late stage of the disease (10, 11). Interestingly, a subsequent study on biopsy specimens found similar conglomerates of lymphocytes in the meninges of MS patients at a very early stage of the disease (20).

**Figure 1:**
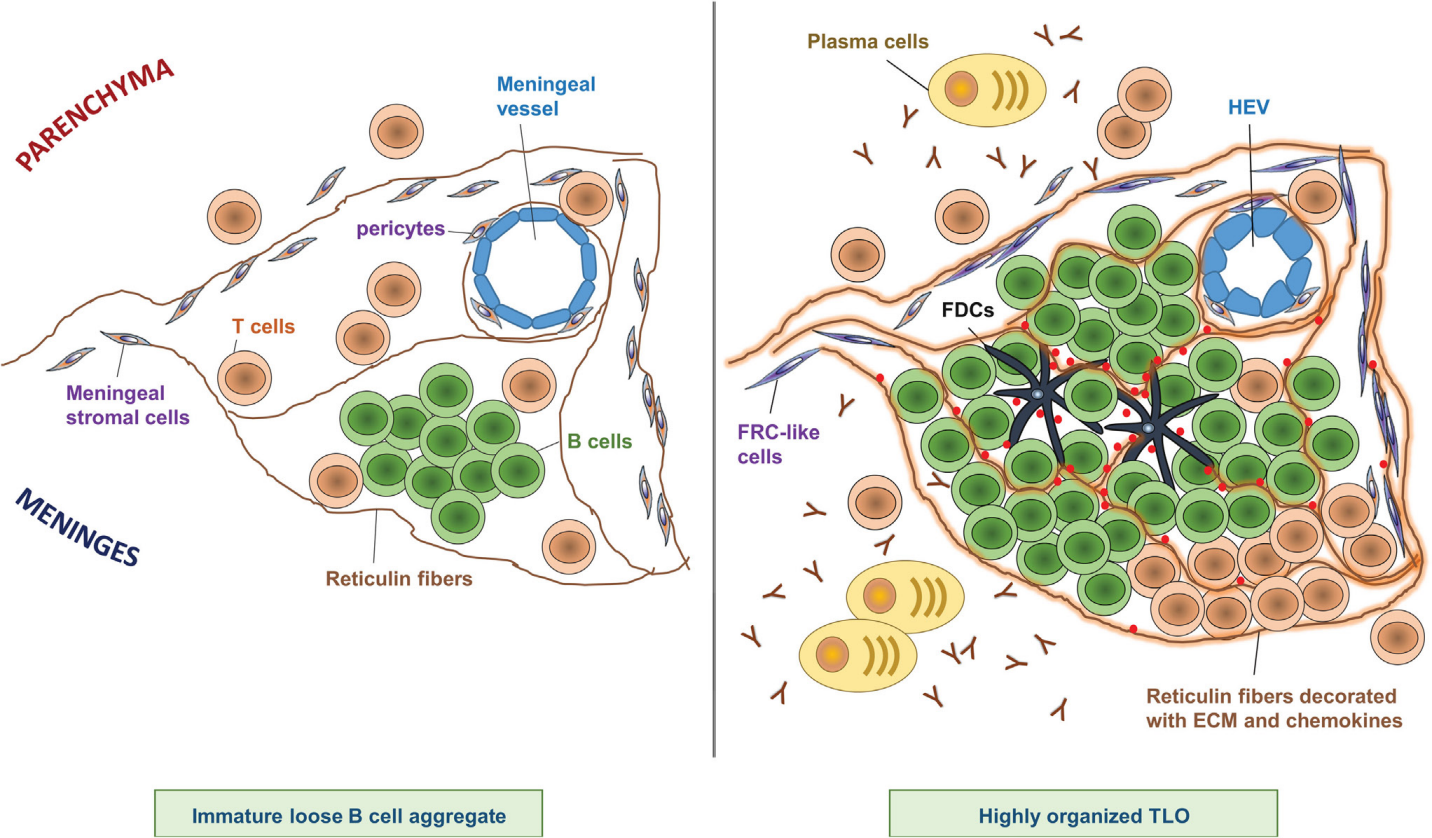
**Composition of CNS TLOs**. TLOs in the CNS show different degrees of organization ranging from immature loose B cell aggregates (left) to highly organized lymphoid structures (right). Notably, at any time, only a minor fraction of cellular aggregates shows the highest degree of organization, however, the frequency of highly organized TLOs increases over time after onset of disease. Immature loose B cell aggregates show clustering of B cells in the meninges often in proximity to a meningeal blood vessel, scattered T cells, and a few reticulin fibers (left panel). In contrast, highly organized TLOs (right panel) are characterized by large B cell clusters next to a more or less defined T cell zone, and a dense network of reticulin fibers decorated with extracellular matrix (ECM) and chemokines. Reticulin fibers are produced by meningeal stromal cells that have differentiated into FRC-like cells. Sometimes FDCs can be detected at the center of the B cell follicle, which may have differentiated from pericytes. Plasma cells and antibodies are found in proximity to highly organized TLOs. In addition, HEVs can be detected in some highly organized TLOs, which may regulate infiltration of lymphocytes into the TLO.

The degree of meningeal inflammation varied between analyzed samples. However, it was shown that an increased level of general perivascular and meningeal inflammation was accompanied by meningeal aggregates of CD20^+^ B cells, CD138^+^ and Ig^+^ PCs, and T cells buried in the sulci of the frontal, temporal, and parietal lobes, and in particular associated with the cingulate and precentral gyrus (10). Meningeal infiltrates exhibited various stages of organization, from elementary clusters of cells to highly organized follicle-like structures (10) (Figure 1). In line with this observation, some B cell aggregates featured a complex reticular network formed by CD35^+^ FDCs (10).

Interestingly, the degree of meningeal inflammation in the forebrain correlated with the degree of cerebellar meningeal inflammation (21). Yet, cerebellar meninges did not exhibit lymphoid-like aggregates as detected in the forebrain. One could argue that specific properties of the cerebellar subarachnoid space restrain the formation of such structures. Furthermore, analysis of cerebellar meninges revealed a high density of CD68^+^ macrophages, and the number of macrophages correlated with parenchymal microglial activation, fitting well with earlier reports that described an association between meningeal cellular infiltrates and parenchymal microglial/macrophage activation (21, 22).

Many B cells within follicles stained positive for the proliferation marker Ki67 (10). B cell aggregates with well-developed FDC networks showed clusters of Ki67^+^ nuclei suggesting formation of germinal centers. However, these follicles lack an interfollicular T cell zone with HEV as it was observed in ectopic lymphoid tissue in other organs.

In conflict to these data, a recent report studying a different cohort of patients did not detect any follicle-like structures in the meninges of MS patients (23).

In summary, these observations suggest that some MS patients exhibit strong meningeal inflammation with formation of well-organized follicles. These intrameningeal structures feature some characteristics reminiscent of TLOs as they have been described in other persistently inflamed organs. Discrepancies in regard to the detection of these aggregates may be based on biological heterogeneity across patients. Furthermore, it is not clear whether these immune cell infiltrates persist for longer periods or whether they are only transiently present during periods of more active CNS inflammation. Technical difficulties may also contribute to this inconsistency as these structures tend to be very small (<100 μm in thickness), and meninges easily detach at autopsy. Therefore, these follicle-like structures may get lost during tissue processing. One challenge might be to provide guidelines on how to handle the tissue and to define criteria that allow the reproducible identification of these structures.

### Induction/Formation of TLOs in MS

As only a fraction of MS patients shows B cell follicles in the meninges, ectopic lymphoid neogenesis must be controlled by a specific set of inflammatory signals. Likewise, it is known that some tissues and tumors are more prone to the development of TLOs than others indicating an important role of the microenvironment. The CNS holds a special status as several barriers, including the blood–brain barrier (BBB), the blood–meningeal barrier and the blood–CSF barrier control passage of macromolecules and immune cell infiltration (24). However, the CNS is rather an immune-specialized than an immune-privileged site, as it is controlled by extensive immune surveillance under physiologic conditions (24). Moreover, recent reports have described a functional lymphatic system in the CNS draining macromolecules and cells to deep cervical lymph nodes (cLN) (25, 26).

The question is what factors induce the formation of ectopic lymphoid follicles in this special environment. It is assumed that the formation of TLOs mimics significant steps of the organogenesis of SLOs. Initiation of SLOs is characterized by a close and reciprocal interaction of hematopoietic CD4^+^CD45^+^CD3^−^ lymphoid tissue inducer (LTi) cells and lymphoid tissue organizer (LTo) cells of mesenchymal origin. The lymphoid chemokine CXCL13 released by LTo cells plays a crucial role as it attracts CXCR5-expressing LTi cells (12). CXCL13 is elevated in CSF and active lesions of MS patients and intrathecal Ig and occurrence of B cells and plasmablasts correlates with CXCL13 levels in the CSF (27–30). Furthermore, about 20% of CSF CD4^+^ T cells and almost all B cells express CXCR5 suggesting that CXCL13 serves as an important chemoattractant to the CNS compartment (27). Moreover, FDCs in meningeal lymphoid aggregates express CXCL13 further indicating an important role for this chemokine in TLO formation (10).

Interestingly, untreated MS patients show elevated numbers of innate lymphoid cells in the peripheral blood and CSF (31, 32), and a recent study detected retinoic acid receptor-related orphan receptor γt (RORγt)-positive and CD3-negative cells in submeningeal B cell follicles (33). These cells may represent group 3 innate lymphoid cells, which comprise the LTi cell subset, and thus could potentially be involved in TLO formation.

In addition, there is evidence that, besides LTis, certain immune cell subsets in inflammatory lesions can trigger the development of TLOs. In particular, IL-17-secreting CD4 T helper (Th17) cells have been implicated in the pathogenesis of TLOs, and MS patients show an increased frequency of Th17 cells in peripheral blood and CSF compared with controls (34–36). Th17 cells secrete a variety of cytokines, among others IL-17, IL-21, and IL-22 (37). Interestingly, all of them have been linked to the formation of lymphoid follicles (18). As IL-17 was shown to induce CXCL13 (38), this might be an additional, indirect mechanism how Th17 cells contribute to the formation of TLOs.

In this context, it is interesting that there might be an ancestral link between adult LTi cells and Th17 cells (39, 40). Indeed, LTi cells and Th17 cells share several features as both cells express the transcriptional regulator RORγt; can respond to IL-23 and aryl hydrocarbon receptor ligands; and can produce IL-17, IL-22, and GM-CSF (41).

These factors and probably others like BAFF (B lymphocyte stimulator) and LT might contribute to create a permissive microenvironment for accumulation of B cells during chronic inflammatory conditions.

### Function of TLOs in MS

Upon antigen encounter, naïve B lymphocytes develop into plasmablasts and undergo further maturation, e.g., hypermutation in germinal centers. TLOs, besides SLOs, are known to provide excellent niches where specific plasmablasts can further differentiate into PCs (12).

Detection of intrathecal Ig synthesis and oligoclonal bands (OCB) are typical features in MS patients. Ig synthesis occurs early in the course of the disease, and once acquired, persists unchanged throughout the disease in the majority of cases. Therefore, it serves as an important supportive diagnostic criterion (42).

Multiple sclerosis lesions often show deposition of antibodies and activated complement bound to disintegrating myelin (43, 44). Interestingly, the antibody repertoire in the CSF reflects Ig transcripts of B cells populating both CSF and brain lesions (45). Clonally related, antigen-experienced B cells are found in the CSF and peripheral blood as well as in the meninges and the parenchyma of MS patients suggesting an active immune axis between these different compartments (45–47). However, it remains unclear where these B cells encounter antigen and mature further. A recent study analyzed paired tissues comprising cLN and CNS by deep sequencing. Founding members of clonal families were primarily detected in cLNs, while more mature members of these founders were found both in the cLNs and the CNS (48). These data provide further evidence that B cells can overcome tissue barriers between the periphery and the CNS. However, they also suggest that while the first antigen contact occurs in the periphery further maturation can happen both in the periphery and the CNS.

Meningeal immune cell aggregates recapitulate lymphoid follicle-like features to some extent and, therefore, may provide an excellent microenvironment for the interaction of B cells with T cells and FDC. Accordingly, they might support local B and T cell activation and further maturation. Therefore, one could speculate that antigen-experienced and clonally expanded B cells arise from these structures.

Overall, the above-mentioned studies suggest a role of meningeal TLOs in humoral- and cell-mediated immunity in MS patients.

### Clinical Relevance of TLOs in MS

It is still not clear whether meningeal TLOs have a pathophysiological significance in MS or just represent an epiphenomenon. A recent postmortem study of chronic MS patients revealed extensive cortical demyelination affecting about 25% of the cerebral cortex in contrast to 5% of the subcortical/periventricular white matter (WM) (49). Similar observations were made in the cerebellum where demyelination of gray matter (GM) was far more pronounced than WM pathology (WM 3% versus GM 14%, representing % area of lesion in relation to total area) (21). Recently, MRI studies further broadened our knowledge about the prevalence of cortical lesions in various subtypes of MS and at different stages of the disease (50). Cortical lesions are clearly linked to cognitive impairment and disability progression (51). In this context, it is interesting that several studies suggested a correlation between the occurrence of meningeal inflammation and cortical demyelination (11, 20–22, 52, 53). Notably, formation of follicle-like structures in a fraction of SPMS cases was accompanied by increased meningeal inflammation and was associated with pronounced subpial cortical pathology. Concurrently, follicle positive cases showed a more severe disease course with younger age at disease onset, younger age at irreversible disability, and earlier death (11, 22). In addition, a study analyzing cortical biopsy specimens of early-stage MS patients also revealed a strong topographic association of moderate-to-marked meningeal inflammation with cortical demyelination (20). However, these findings are still a matter of debate and need validation in larger cohorts, especially since another study did not find evidence for an association of meningeal inflammation and cortical lesions in chronic MS (23).

Further histological analysis of follicle positive SPMS cases revealed a significant reduction in the thickness of the cortical GM layers in the precentral, frontal, and temporal gyrus (52). In line with this observation, these cases exhibited a substantial loss of neurons, which also included neurons with a pyramidal morphology. Interestingly, these pathologic changes exhibited a clear pial to WM gradient in the precentral gyrus, both in GM lesions and normal appearing GM (52). Overall, these observations strengthen the hypothesis that the meningeal inflammatory milieu is involved in the pathogenesis of cortical damage in a substantial number of SPMS cases. CD8^+^ T cell-mediated immunopathology could be a potential cause as follicles harbor a considerable number of CD8^+^ T cells (54). Both direct mechanisms *via* cytotoxic tissue damage and indirect mechanisms, e.g., by inducing activation of microglia might play a role. Other possible triggers are soluble factors released by inflammatory cells in the meninges. Finally, it could also be an antibody-mediated process, as an association between intrathecal immunoglobulin levels and cortical lesion load in patients with clinically isolated syndrome has been reported (55).

A striking difference between WM and GM damage is the lack of inflammatory cell infiltrates and rare deposition of immunoglobulin in cortical lesion (56–58). However, experimental models have shown that the GM does not support the persistence of inflammatory cells over extended periods of time (58). Thus, lack of inflammatory cells in GM with axonal damage or neuronal loss does not necessarily mean that these pathologic changes are not due to prior inflammatory events. However, an alternative hypothesis is that neurodegenerative processes unfold independently of inflammation and contribute to the attrition of GM structures in longstanding MS cases (59). Yet, axonal damage and neuronal loss in GM structures may also be a consequence of distant underlying WM lesions, e.g., *via* Wallerian degeneration. However, there was no correlation between the number of subpial GM lesions and WM lesions suggesting that inflammatory meningeal lesions actually determine GM damage (52).

Eventually, beyond further analysis of tissue samples from MS patients or autopsy tissue, advanced imaging technologies will contribute to solving these questions. In particular, development of MRI techniques that resolve meningeal inflammatory lesions and enable the unequivocal visualization of cortical lesions are sorely needed to analyze these issues in living patients.

Overall, the clinical relevance of meningeal TLOs in MS patients remains elusive. Validity of studies in human samples is limited as most of the tissues available are collected at a late stage of the disease. Poor quality of tissue, i.e., due to a long postmortem interval, might be another handicap. Thus, in order to further our understanding of CNS TLO formation, function, and impact, we can make use of the animal model for MS, EAE.

## Occurrence and Significance in EAE

Experimental autoimmune encephalomyelitis has been employed for decades to study cellular and molecular pathogenic mechanisms that may also be relevant for MS pathogenesis and, in fact, many important mechanistic insights as well as successful therapeutic approaches have emerged from EAE studies. Thus, the EAE model was instrumental in demonstrating the importance of myelin-reactive CD4 T helper cells as disease drivers, as disease can be induced in healthy animals solely by transfer of these cells (60). Furthermore, the encephalitogenic properties of different T helper cell subsets were defined in numerous EAE studies, starting in the 1990s when IFN-γ-producing Th1 clones were described to be pathogenic while Th2 cells were characterized as non-pathogenic in the context of autoimmune CNS inflammation (61–64). When Tregs and Th17 cells entered the stage these studies were revisited and extended to show that both Th1 and Th17 cells can induce EAE, whereas Tregs aim to control the inflammatory processes (65). Since the majority of research efforts in the EAE field focused on T helper cells, the efficacy of B cell depleting therapies in MS came as quite a surprise for EAE researchers and raised the question why the obviously pathogenic role of B cells in the disease process was not recognized earlier in the EAE model. Rather than neglect and ignorance of the investigators, the most important reason lies in the experimental details of the model itself: the majority of EAE studies use immunization with myelin peptides together with adjuvant to induce disease. Since the self-peptide is already provided and, in this form, will most efficiently be taken up and presented by DCs, a potential role of B cells in processing/presentation of self-antigen and thereby activation of self-reactive T cells is largely bypassed in peptide immunization models. As a prominent example, it was shown that development of disease in C57Bl/6 mice immunized with MOG_35–55_ in CFA – the most widely used EAE model – is B cell independent and features very limited humoral responses (66, 67), whereas immunization of C57Bl/6 mice with recombinant human MOG protein relies on B cells to process and present the antigen and initiate the pathogenic cascade (66, 68, 69). Hence, moving away from the EAE blockbuster model, one can find several different EAE models in the literature that involve strong pathogenic (and also regulatory) B cell responses. Besides studying the role of B cells as antibody sources, APCs, and cytokine producers, a few studies have also described B cells to participate in TLOs in the CNS of EAE mice:

- After description of TLOs in MS brain, TLOs in EAE were first searched for and identified with relatively low frequency in SJL mice immunized with proteolipid protein (PLP)_139–151_ – the only model, where TLOs develop after immunization with peptide (70, 71). In contrast, there is no robust evidence for TLO formation in the classical C57Bl/6-MOG_35–55_ immunization model. One reason for this difference may lie in the genetic background, since in the RRMS of the SJL background TLOs have more time to develop than in the relatively short acute–chronic disease course following immunization of C57Bl/6 mice with MOG_35–55_.
- TLOs have also been described in C57Bl/6 mice immunized with a MBP–PLP fusion protein (MP4). In this model, mice develop a B cell-dependent chronic EAE that can be observed for up to 60 days post immunization (72).
- In the opticospinal EAE (OSE)-mouse, which features both a transgenic MOG-specific TCR as well as a MOG-specific BCR and spontaneously develops EAE, TLO-like structures were found in the CNS (73, 74).
- Following adoptive transfer of either MOG-specific Th17 cells in the C57Bl/6 background or PLP-specific Th17 cells in the SJL background, TLOs were identified with relatively high frequency in the CNS of sick mice (75, 76).

The fact that TLOs have been detected in several models that differ in regard to genetic background, identity and form of antigen, and method of disease induction implies that TLO formation is not just an exotic epiphenomenon, but will occur under varying conditions. However, characterization of EAE TLOs yielded very heterogeneous results not only between different models but also within the same model, suggesting that TLO development and regulation is very complex. Below, we are summarizing the results from different models regarding location, structural and cellular composition, induction, function, and clinical relevance of EAE TLOs to evaluate whether general principles important for CNS TLO formation can be identified, which could then also be applicable for MS TLOs.

### TLO Location and Composition in EAE

**Figure 2:**
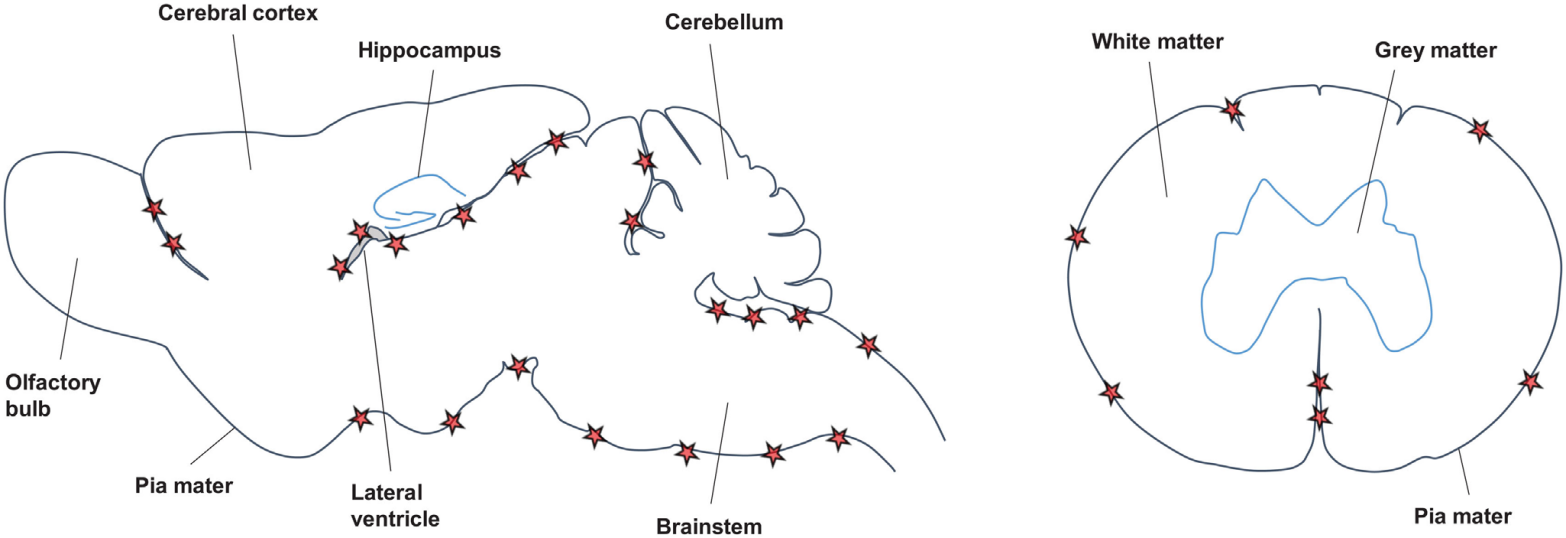
**Location of CNS TLOs**. Sagittal view of the mouse brain (left) and horizontal view of the spinal cord (right) show preferential location of CNS TLOs (red stars) in association with the meninges.

Across the different EAE models and in agreement with the data from MS studies (see Features/Characteristics of TLOs in MS), TLOs are consistently found in association with meninges (Figure 2). Thus, both in the SJL immunization model as well as after transfer of Th17 cells on the SJL background, TLOs form primarily in the brain meninges, particularly those lining brainstem and ventricles (70, 71, 76). Similarly, in the MP4 immunization model TLOs form in the cerebral periventricular space, but also in the cerebellum and in association with spinal meninges (72). Upon transfer of Th17 cells in the C57Bl/6 background the majority of TLOs are located in the spinal meninges (75), and in the spontaneous OSE model TLOs are exclusively found in the spinal meninges (74). Whether TLOs form in the brain or spinal cord is most likely a consequence of the main route of infiltration, which differs between models. Thus, it has been shown that in the OSE model inflammation is restricted to optic nerve and spinal cord (73, 77). In addition, it is known that Th17 cells promote brain inflammation (78). However, whether in the brain or in the spinal cord, it is clear that TLOs preferentially form in association with meninges. This may be partly due to the high density of vessels in the meninges compared with the parenchyma, which have been shown to be an important point of entry for infiltrating leukocytes (79, 80). HEV, which express adhesion molecules like PNAd or MadCAM to specifically attract naïve lymphocytes into SLOs and which have also been described in TLOs in other organs, were detected within TLOs in the MP4-immunization model (72). However, no evidence for HEVs was found in the spontaneous OSE model nor in the Th17 transfer model (C57Bl/6 background) (74, 75), while presence of HEVs was not determined in the SJL immunization and SJL Th17 transfer models. Since HEVs develop in response to cytokines like LT, their formation may require some time and occur only in the most mature TLOs. In addition, it is also possible that due to the abundance of meningeal vessels, which become permeable during CNS inflammation due to break-down of the BBB, the development of HEVs is not absolutely required for the recruitment of lymphocytes into CNS TLOs. In contrast, for example, tumor TLOs may be much more dependent on proper development of HEVs since the tumor environment is comparatively poorly vascularized.

Another important reason for the preferred formation of TLOs in association with meninges is the structural support that can be provided by the meningeal stromal cells. SLOs are structurally organized by fibroblastic reticular cells (FRCs), which line the subcapsular sinus and the conduits for antigen transport into the T cell zone, and secrete ECM components to form a dense network of reticulin fibers along which T cells, APCs, and B cells can migrate to their designated areas. FRCs are also an important source of the chemokines that retain T cells (CCL19 and CCL21) and B cells (CXCL13) in the SLO and organize it into separate B cell and T cell zones. In EAE TLOs, reticulin fibers suggestive of stromal FRC-like cells were described in several models, including the MP4-immunization model, the spontaneous OSE model, and the Th17 transfer model (C57Bl/6) (72, 73, 75). In a more detailed analysis, Pikor and colleagues could show that the fraction of FRC-like meningeal fibroblasts, which express Pdpn, PDGFRα, PDGFRβ, and Cadherin11 on their surface and secrete ECM components including fibronectin and ERTR7, increases during CNS inflammation (76). Therefore, the relative abundance of fibroblasts in the meninges, which can be stimulated to adopt FRC-like phenotype and function, may partly explain why TLOs are predominantly located in the meninges.

The other structurally essential stromal cell in the SLO is the FDC, which is positioned in the B cell follicles, where it presents opsonized antigen to induce affinity maturation of B cells and produces a network of reticulin fibers organizing migration into and structure of the follicle. In addition, FDCs are the main source of the B cell attracting chemokine CXCL13 in SLOs. Data from the different EAE models are heterogeneous in regard to the presence of FDCs in CNS TLOs. FDCs could not be identified in the spontaneous OSE model, nor in the Th17-transfer models (both C57Bl/6 and SJL), whereas FDCs were detected *via* the complement receptor CD35 or the marker FDC-M1 in the SJL- and the MP4-immunization model (71, 72). Like FRCs, FDCs are of stromal origin, and it has been suggested that they appear in TLOs as a consequence of differentiation/maturation of ubiquitous local precursors, namely PDGFRβ^+^ pericytes (81), rather than migrating from SLOs. This maturation process requires the proper cytokine signals (primarily LT, TNF and potentially also others) and it may also take some time. Consistent with this idea, FDC-positive TLOs were observed late in the disease course, either upon relapses in the SJL-immunization model or in the chronic phase of EAE 30–57 days post onset in the MP4-immunization model (71, 72). Thus, absence of FDCs in the spontaneous OSE model and Th17 transfer models might be caused by lack of appropriate FDC maturation signals or by lack of time leading to an incomplete differentiation process. The latter may be especially true for the Th17 transfer models, where mice were analyzed comparatively early (5–20 days post onset) to prevent extended suffering due to severity of disease (75, 76). On the other hand, it is also unclear whether FDCs are required for antigen presentation in germinal center reactions in TLOs. Though presence of FDCs has been demonstrated in TLOs in other organs, it remains to be formally shown that they are required for antigen presentation. Thus, it is possible that other APCs can substitute for FDCs in CNS TLOs. Considering that meningeal macrophages have been shown to present antigen and reactivate infiltrating T cells during EAE (79, 80, 82, 83) and that they can be easily detected scattered in and around CNS TLOs they are certainly a good candidate.

Aside from structural components, segregated T and B cell zones are of course a hallmark characteristic for SLOs and TLOs. Across all EAE models, aggregation of B cells was considered a defining criterion for CNS TLOs. In contrast, T cells seemed to be scattered rather than clustered and were detected around and sporadically within the B cell zones (Figure 1). Their presence on the border or within the B cell zone may be a consequence of T cells acting as follicular T helper cells (TFH) to support the B cell maturation process (see TLO Function in EAE). Furthermore, the chemokines responsible for formation of a separate T cell zone (mainly CCL19 and CCL21) may not be so strongly expressed in the CNS, in contrast to the B cell zone-organizing chemokine CXCL13, which has been detected in several of the EAE models (see Induction/Formation of TLOs in EAE). Given that reactivation of T cells in the CNS and especially in the meninges has been clearly demonstrated also in the absence of TLOs (79, 80, 82, 83), T cells may also be less dependent on structure and clustering for their reactivation than B cells.

### Induction/Formation of TLOs in EAE

For the formation of SLOs both LTo and LTi are required. During normal lymph node development, LTis (CD4^+^CD3^−^RORγt^+^IL7Rα^+^) stimulate stromal cells to differentiate into LTos, which in turn start producing fibers and conduits and expressing adhesion molecules and chemokines that organize the SLO and guide lymphocytes to their designated areas. This stimulation of LTos by LTis is mediated primarily *via* cytokines of the TNF superfamily, especially LT. The heterotrimer LTα_1_β_2_ expressed on the LTi cell surface engages with LTβ receptors on stromal cells to initiate the differentiation process. Additionally, LTis also secrete cytokines, including LTα_3_, and TNFα which further activate LTos. In TLO formation, other cell types including type 3 innate lymphoid cells and T cells can perform the tasks of LTi, as long as they can provide the proper cytokine signals (84). Which cell type initiates the TLO formation process in the CNS was not investigated in detail in the immunization models nor in the spontaneous OSE model. However, increased expression of LT was detected in the SJL-immunization model at onset and upon relapses, and neutralization of LT prevented relapses and decreased expression of the B cell attracting chemokine CXCL13 in the CNS (71). These data suggested that LT-signaling is involved in activating LTos in the CNS, but did not identify the cellular source of LT. The Th17 adoptive transfer model (C57Bl/6) demonstrated that Th17 cells themselves are the cellular initiators for CNS TLO formation, since TLO formation was not observed after transfer of other T cell subsets, including Th1 cells, despite development of clinical disease (75, 85). Interestingly, Th17 cells and classical LTi share expression of several markers, including RORγt, IL-17, IL-22, CCR6, and IL-7Rα, and thus Th17 cells may also be able to function as LTi in stimulating CNS fibroblasts to act as LTo. This hypothesis was investigated in detail in the SJL Th17 adoptive transfer model (76): Pikor and colleagues could show that IL-17 and IL-22 together act on meningeal FRCs to induce remodeling of actin and collagen and production of ECM components, which ultimately leads to formation of fibrous networks providing proper structure for the TLO. Interestingly, IL-17 and IL-22 also induced expression of IL-6 and IL-23 in FRCs, which in turn supported *de novo* Th17 differentiation in the CNS, and may be a mechanism to ensure stable supply of Th17 cytokines for the FRCs to maintain their differentiation status. While FRC remodeling was independent of LT-signaling, production of CXCL13 by meningeal FRCs and therefore accumulation of B cells and deposition of complement in the lesion required intact LT-signaling. Together, these data suggest that the combination of LT and the Th17 cytokines IL-17 and IL-22 efficiently stimulates the meningeal fibroblasts to differentiate into FRCs (and potentially FDCs) and act as LTo in CNS TLOs. This also explains why there is almost no TLO formation in recipients of pure Th1 cells (75): although Th1 cells produce LT and TNFα and thus may even activate the meningeal fibroblast and stimulate them to produce CXCL13, complete maturation into FRCs (and potentially FDCs) additionally requires the presence of the Th17 cytokines IL-17 and IL-22. Importantly, both IL-17 and IL-22 have also been implicated in TLO formation in other organs/disease models including during iBALT formation in the lung (38), lymphoid follicle formation in the intestine during *Citrobacter* infection (86), and virus-induced TLO formation in salivary glands (87).

Data regarding the impact and requirement of chemokines for CNS TLO formation in EAE are limited. As already mentioned, CXCL13 is produced primarily by FDCs in SLOs to attract CXCR5-expressing B cells and TFH cells into the follicles and form a separate B cell zone. In all EAE models except the OSE model elevated CXCL13 mRNA levels were reported, and in some cases CXCL13 protein was also detected by immunohistochemistry at least in the more mature TLOs. CXCL13 was detected even in the absence of FDCs suggesting that in the CNS other cells can be a significant source of this chemokine. In fact, Pikor and colleagues showed that meningeal FRCs can produce CXCL13 in response to LT (76), and a recent study demonstrates that FRCs can also produce CXCL13 in response to IL-22 (87). While the evidence for presence of CXCL13 in CNS TLOs is comparatively robust, evidence that CXCL13 actually causes the attraction and aggregation of B cells into CNS TLOs is still missing. Another chemokine that may play a role in CNS TLO formation is CXCL12, which interacts with CXCR4^+^ B cells. Intriguingly, Fleige and colleagues have shown that IL-17 can induce CXCL12 expression in stromal cells leading to FDC-independent TLO formation in the lung during *P. aeruginosa* infection (88), and another study showed that IL-22 can upregulate CXCL12 expression in epithelial cells (87). Given the importance of Th17 cells for CNS TLO formation in EAE, it is plausible that CXCL12 may contribute to B cell attraction and aggregation. While CXCL12 has been detected in standard EAE models, where it was suggested to retain infiltrating cells in the perivascular compartment (89), presence/relevance of CXCL12 was not tested in any of the TLO EAE models. The role of T cell chemokines for formation of CNS TLOs in EAE is even less understood. In SLOs, FRCs produce CCL19 and CCL21 to guide CCR7-positive T cells into the T cell zone. Constitutive expression of CCL19 has been detected in healthy CNS and increased levels of CCL19 and CCL21 were detected in the CNS in regular EAE and MS (90–92). The SJL Th17 adoptive transfer model showed a slight LT-independent upregulation of CCL21 in meningeal FRCs (76), but it remains to be determined whether this is relevant for CNS TLO formation. Taken together, the data suggest that CCL19/CCL21 produced by meningeal FRCs may recruit T cells into CNS TLOs in EAE. However, as mentioned already above, the fact that T cells seem to be much more scattered than B cells in CNS TLOs questions the presence and also the need for strong T cell chemokine signals that establish formation of a separate T cell zone. In summary, much more functional experimental work is needed to clearly define the role of chemokines in CNS TLO formation.

### TLO Function in EAE

As for SLOs, the functions of a fully developed TLO comprise priming of naïve lymphocytes to new locally derived antigen resulting in differentiated antigen-specific T effector cells and B cells that underwent germinal center reactions yielding affinity-matured B memory cells and antibody-producing PCs. Studies in the MP4-immunization model, the spontaneous OSE model, and the SJL Th17 adoptive transfer model revealed T cell proliferation in TLOs (72, 74, 76). However, since T cells can also proliferate in the CNS in the absence of TLOs these data alone do not prove functionality of CNS TLOs. Additional evidence is provided in the MP4-immunization model, since T cells isolated from the CNS showed specificity not only for the immunizing antigen MP4 but also for the CNS-specific antigen MOG (72), suggesting that those MOG-specific T cells may have been primed directly in CNS TLOs. Although one cannot completely exclude the possibility that these MOG-specific T cells were primed in the periphery, the fact that MOG-specific T cells could not easily be detected in SLOs points toward T cell priming in the CNS. Similarly, in the SJL Th17 adoptive transfer model it was shown that in order to detect endogenous T cells differentiated into Th17 effector cells in the CNS, transferred T cells needed to express LT, which in turn stimulated meningeal FRC remodeling and expression of Th17 differentiation cytokines (76). These data – though not definite evidence – still support the idea that priming and differentiation of naïve T cells in response to CNS antigen may happen in CNS TLOs; however, more experimental evidence is needed to confirm this hypothesis. In regard to germinal center reactions, it was demonstrated in the C57Bl/6 Th17 adoptive transfer model that transferred Th17 cells in the CNS expressed markers associated with TFH cells including CXCR5 and Bcl6, as well as GC markers like GL7 and PNA (75). Thus, Th17 cells may function as TFH cells in CNS TLOs and provide help to B cells in GC reactions. In line with this, Th17 cells were shown previously to be excellent B cell helpers (93), and thus, the plasticity of Th17 cells to adopt TFH functions may be another reason why Th17 cells are superior to other T cell subsets in supporting TLO formation. Accordingly, some but not all CNS B cells also expressed GC markers including GL7, PNA, and Bcl6, some had undergone class switch recombination to IgG, and a few PCs were detectable in the CNS of Th17 recipients (75). Proliferating B cells were also found in the SJL and MP4-immunization model (70–72), and some B cells in the MP4-immunization model also expressed GC markers and AICD, the enzyme required for CSR and somatic hypermutation (72, 94). Consistent with this finding a germline transcript analysis indicated that B cells switched to IgG2b and IgG3 in the CNS, and sequencing of Ig transcripts revealed some CNS B cell clones that could not be detected in the spleen (94). Although these data support the hypothesis that GC reactions may happen in CNS TLOs, one has to keep in mind that it is extremely difficult to exclude that lymphocytes acquired the observed phenotype in the periphery and then migrated to the CNS. In contrast to these data, CNS B cells in the spontaneous OSE model showed no GC phenotype nor isotype switch, but appeared to be rather activated naïve B cells (74), confirming the notion that across the different models CNS TLOs are quite heterogeneous in their stage of development (Figure 1). Together the data from the different models suggest that definitely not all, but maybe the most organized and mature TLOs are also functional in supporting generation of differentiated T effector cells and affinity-matured B cells specific to CNS antigens.

### Clinical Relevance of TLOs in EAE

Although CNS TLOs have been detected in several EAE models, their impact on the clinical disease course is completely unclear. Based on data from MS patients, where occurrence of TLOs was associated with a more aggressive disease course (10, 11), it has been postulated that TLOs in the CNS support differentiation and maturation of CNS antigen-specific effector lymphocytes, which continuously fuel the inflammatory process and thereby drive disease progression and chronicity. Except for the spontaneous OSE model, where TLO frequency and size was associated with a chronic and more severe disease course (74), presence of TLOs did not clearly correlate with disease severity in any of the other TLO EAE models. This may be explained by TLO frequency and kinetics of TLO development: In the SJL and MP4-immunization models TLO frequency increases in the late phases of disease, i.e., upon relapses or in the chronic phase of EAE 30–57 days post onset (71, 72). Although these late time points allow the TLOs to become comparatively mature and organized, the overall TLO frequency may still be too low (around 10 TLOs per mouse) to have a clinically visible effect. On the other hand, TLO frequency is significantly higher in the Th17 adoptive transfer models (around 60 TLOs per mouse) than in immunization models; however, mice were analyzed relatively early (2–15 days post onset) and thus TLOs may not yet be developed enough (reflected by lack of FDCs and HEVs) to affect the disease course (75, 76). In addition, the disease severity in Th17 adoptive transfer EAE is generally very pronounced and mice often show no recovery possibly due to irreversible tissue damage, making it very difficult to detect an exacerbation of clinical signs, especially using the rough scoring system common in the EAE field. Although it is likely, that CNS TLOs worsen clinical disease, especially considering that in the SJL Th17 adoptive transfer model TLOs promoted differentiation of endogenous Th17 effector cells (76), there is also the possibility that CNS TLOs counteract inflammation by generating regulatory T and B cells. This phenomenon has been observed in TLOs in the tumor microenvironment, but has never been investigated in CNS TLOs in EAE. A correlation of TLOs with cortical pathology, as described in MS brain (see Clinical relevance of TLOs in MS), has so far not been detected in TLO EAE models. Considering that cortical pathology may be caused by soluble and/or cellular factors emerging from meningeal TLOs over time, lack of cortical pathology in EAE may be a consequence of the short disease course when compared to human MS. On the other hand, it is also possible that this pathologic aspect of MS is simply not well recapitulated in EAE, since important cues (for example, presence and action of CD8 T cells) may be different in EAE and MS.

In order to define the clinical relevance of TLOs, an EAE model with reasonable frequency of TLOs and relatively mild disease course, which can be observed for long time periods, would be ideal. In addition, experimental methods need to be developed, which inhibit TLO formation in the CNS without disturbing the regular immune responses happening in the CNS and in the periphery during EAE. Thereby, one could gain detailed insight into the clinical relevance of TLOs in EAE.

## Concluding Remarks

In summary, data from the last decade have revealed the existence of TLOs in the CNS of MS patients, and TLO formation can also be recapitulated in the MS animal model EAE, which will enable us to study the mechanisms of TLO formation as well as their precise functions and impact on disease development in more detail in the future. In general, the existence of TLOs further support an important role of B cells in MS pathogenesis, but the data also show a wide spectrum of TLO developmental stages ranging from simple B cell aggregates to highly organized structures (Figure 1), suggesting that a complex network of cellular players and cytokine signals is required to build these structures. Although some contributing factors and mechanisms like involvement of Th17 cells have now been exposed, considerable experimental work is needed to understand the phenomenon of CNS TLOs. The hypothesis that CNS TLOs support differentiation and maturation of CNS antigen-specific T and B effector cells, and thereby propagate continuous inflammation directly in the CNS – although attractive – remains to be first tested experimentally, and only then one can start thinking about CNS TLOs as therapeutic targets.

## Author Contributions

AP and MM contributed equally to the writing of this review article.

## Conflict of Interest Statement

The authors declare that the research was conducted in the absence of any commercial or financial relationships that could be construed as a potential conflict of interest.
